# Laying the Foundation for Crassulacean Acid Metabolism (CAM) Biodesign: Expression of the C_4_ Metabolism Cycle Genes of CAM in *Arabidopsis*

**DOI:** 10.3389/fpls.2019.00101

**Published:** 2019-02-11

**Authors:** Sung Don Lim, Sojeong Lee, Won-Gyu Choi, Won Cheol Yim, John C. Cushman

**Affiliations:** Department of Biochemistry and Molecular Biology, University of Nevada, Reno, Reno, NV, United States

**Keywords:** crassulacean acid metabolism, CAM biodesign, water-use efficiency, C_4_ metabolism, ice plant, *Mesembryanthemum crystallinum*, *Arabidopsis thaliana*

## Abstract

Crassulacean acid metabolism (CAM) is a specialized mode of photosynthesis that exploits a temporal CO_2_ pump with nocturnal CO_2_ uptake and concentration to reduce photorespiration, improve water-use efficiency (WUE), and optimize the adaptability of plants to hotter and drier climates. Introducing the CAM photosynthetic machinery into C_3_ (or C_4_) photosynthesis plants (CAM Biodesign) represents a potentially breakthrough strategy for improving WUE while maintaining high productivity. To optimize the success of CAM Biodesign approaches, the functional analysis of individual C_4_ metabolism cycle genes is necessary to identify the essential genes for robust CAM pathway introduction. Here, we isolated and analyzed the subcellular localizations of 13 enzymes and regulatory proteins of the C_4_ metabolism cycle of CAM from the common ice plant in stably transformed *Arabidopsis thaliana*. Six components of the carboxylation module were analyzed including beta-carbonic anhydrase (*McBCA2*), phosphoenolpyruvate carboxylase (*McPEPC1*), phosphoenolpyruvate carboxylase kinase (*McPPCK1*), NAD-dependent malate dehydrogenase (*McNAD-MDH1*, *McNAD-MDH2*), and NADP-dependent malate dehydrogenase (*McNADP-MDH1*). In addition, seven components of the decarboxylation module were analyzed including NAD-dependent malic enzyme (*McNAD-ME1*, *McNAD-ME2*), NADP-dependent malic enzyme (*McNADP-ME1*, *NADP-ME2*), pyruvate, orthophosphate dikinase (*McPPDK*), pyruvate, orthophosphate dikinase-regulatory protein (*McPPDK-RP*), and phosphoenolpyruvate carboxykinase (*McPEPCK*). Ectopic overexpression of most C_4_-metabolism cycle components resulted in increased rosette diameter, leaf area, and leaf fresh weight of *A. thaliana* except for *McNADP-MDH1*, *McPPDK-RP*, and *McPEPCK.* Overexpression of most carboxylation module components resulted in increased stomatal conductance and dawn/dusk titratable acidity (TA) as an indirect measure of organic acid (mainly malate) accumulation in *A. thaliana*. In contrast, overexpression of the decarboxylating malic enzymes reduced stomatal conductance and TA. This comprehensive study provides fundamental insights into the relative functional contributions of each of the individual components of the core C_4_-metabolism cycle of CAM and represents a critical first step in laying the foundation for CAM Biodesign.

## Introduction

Crassulacean acid metabolism (CAM) is a temporally controlled, inorganic carbon-concentrating mechanism that improves water-use efficiency (WUE) by shifting all or part of CO_2_ uptake from the day to the night when air:leaf water vapor pressure deficits are lower compared with the day (Griffiths, 1989). CAM is distinguished from C_3_ and C_4_ photosynthesis by temporally separating the primary carbon fixation by phospho*enol*pyruvate (PEP) carboxylase (PEPC) using HCO_3_^-^ and the secondary fixation phase by ribulose-1, 5-bisphosphate carboxylase/oxygenase (RUBISCO) using CO_2_, which are linked by organic acid (mainly malate) storage intermediates. Cytosolic PEPC activity in cooperation with NAD(P)-malate dehydrogenase (NAD(P)-MDH) leads to nocturnal CO_2_ uptake and the formation of malate production and storage in the vacuole. Nocturnal CO_2_ uptake by PEPC is made possible by inverse stomatal behavior, in which stomata are open at night, but closed during all or part of the day, thereby reducing water loss from the plant. The C_4_ acids accumulated overnight are subsequently decarboxylated during the day by either NAD(P)-malic enzyme (ME) to release pyruvate and CO_2_ and pyruvate orthophosphate dikinase (PPDK) to regenerate the pyruvate to PEP or NAD(P)-malate dehydrogenase (MDH) and PEP carboxykinase (PEPCK) to release CO_2_ and regenerate PEP, depending on the species (Winter, 1985; Christopher and Holtum, 1996; Kondo et al., 2000). The CO_2_ is then refixed by chloroplastic RUBISCO, leading to carbohydrate production *via* the Calvin–Benson cycle. This intracellular release of CO_2_ in the vicinity of RUBISCO results in elevated (2- to 60-fold) CO_2_ concentrations within the leaf compared with atmospheric CO_2_ levels (Lüttge, 2002). This ‘CO_2_ pump’ favors RUBISCO’s carboxylase rather than oxygenase activity, which reduces photorespiration, which can reduce the efficiency of photosynthesis up to 40% in C_3_ plants (Ehleringer and Monson, 1993). The net result of the CAM cycle is a 3- to 6-fold improvement in WUE compared with C_3_ and C_4_ photosynthesis species, respectively, and an enhancement of the magnitude and duration of net CO_2_ uptake over a 24-h cycle in resource-limited environments (Borland et al., 2009, 2011).

Introduction of CAM enzymatic machinery into C_3_ and C_4_ photosynthesis crops has been proposed as a potentially useful approach for improving the WUE of these crops (Borland et al., 2014, 2015; DePaoli et al., 2014; Yang et al., 2015). This ambitious goal, referred to as CAM Biodesign, involves the design-build-test-learn iterative cycles of synthetic biology (Nielsen and Keasling, 2016), necessitated by the relative complexity of the CAM pathway and our incomplete understanding of circadian regulatory processes associated with CAM. However, detailed knowledge of the functions of the enzymatic, transport, and regulatory components is required prior to engaging in CAM Biodesign efforts. Such information is also critical for creating and refining metabolic flux balance analysis models of CAM (Cheung et al., 2014) and performing computational analyses of the productivity potential of CAM and engineered CAM (Shameer et al., 2018). To this end, facultative CAM plants provide a useful means of determining precisely which gene family members are recruited to function in CAM (Cushman et al., 2008; Winter and Holtum, 2014; Hartwell et al., 2016).

The common ice plant, *Mesembryanthemum crystallinum*, is an intensively studied facultative CAM model species, in which C_3_ photosynthesis-performing plants can switch to CAM in response to salinity or water-deficit stress (Bohnert and Cushman, 2000; Winter and Holtum, 2014). Upon removal of the stress, the plants revert to C_3_ photosynthesis (Vernon et al., 1988; Kholodov et al., 2004; Nosek et al., 2018). Comparison of the differential patterns of enzymatic activities and protein abundance between the C_3_ photosynthesis and the CAM states has been extremely useful for determining enzymes and transporters that are essential to the performance of CAM (Winter et al., 1982a,b; Paul et al., 1993). More recent studies comparing differential mRNA abundance changes using C_3_/CAM comparisons have allowed for the facile identification of genes encoding key CAM-specific enzymes (Cushman et al., 2008), associated intracellular transporters (Häusler et al., 2000; Kore-eda et al., 2005; Kore-eda et al., 2013), and salt-stress responsive mRNA expression patterns in roots (Tsukagoshi et al., 2015) and epidermal bladder cells (Oh et al., 2015). Proteomic analyses have also revealed differential protein abundance changes of CAM-related enzymes in various cell types and subcellular fractions triggered by salinity stress treatment (Barkla et al., 2012, 2016; Cosentino et al., 2013).

Once the specific genes for CAM are identified, an important prerequisite for CAM Biodesign is a detailed understanding of the subcellular localization of these gene products. Previous studies of the subcellular characterization of CAM enzymes has relied upon subcellular fractionation in several CAM species including *Sedum praealtum* (Spalding et al., 1979), *Bryophyllum calycinum* and *Crassula lycopodioides* (Schnarrenberger et al., 1980), and *M. crystallinum* (Winter et al., 1982a). Alternatively, immunolocalization using enzyme-specific antibodies was used to define the subcellular localization of enzymes from various CAM species (Kondo et al., 1998, 2000; Lara et al., 2004). Subcellular fraction studies suffer from cross-contamination of subcellular compartments or inaccurate results depending upon the fractionation method used. Immunolocalization methods depend upon high-quality antibodies and enough abundance of the target protein for reliable detection. Because of these limitations, the definitive subcellular localization of many other CAM-specific enzymes and transporters have not been defined to date.

In addition to defining the subcellular localization of CAM-related gene products, the individual contribution of each component of the CAM pathway must be understood. One useful approach to understand the function of each CAM gene product is the creation of systematic knock-out or knock-down mutants of key CAM genes. This approach has been used successfully in the obligate CAM model *Kalanchoe fedtschenkoi*, for which a reliable and robust transformation system is available (Hartwell et al., 2016). Knockdown of the activity of either mitochondrial NAD-ME, or cytosolic/plastidic PPDK, dramatically reduced CAM performance, reduced the activity of other CAM enzymes, particularly PEPC, and reduced the circadianly controlled phosphorylation of PEPC by PPCK (Dever et al., 2015). Knockdown of these two enzymes also disrupted or dampened the circadian rhythmic expression of PEPC phosphorylation and PEPC kinase transcript accumulation, and CAM CO_2_ fixation patterns (Dever et al., 2015). Interestingly, knockdown of mitochondrial NAD-ME also disrupted the rhythmic transcripts of core circadian clock genes suggesting that CAM perturbation also disrupts the central circadian clock itself. Knockdown of NADP-ME did not have the same effect, suggesting that NAD-ME is the major decarboxylation enzyme of CAM in this species. Knockdown of PPCK, which is the dedicated regulatory protein kinase of PEPC, resulted in reduced or no detectable nighttime phosphorylation of PEPC and up to a 66% reduction in nocturnal CO_2_ fixation (Boxall et al., 2017). PPCK disruption also resulted in reduced malate accumulation at dawn and reduced nocturnal starch turnover. Loss of PPCK expression also perturbed the expression of many core circadian clock genes, again suggesting that loss of CAM function also perturbs the central circadian clock. These results demonstrate the importance of PPCK in prolonging PEPC activity throughout the night period in *K. fedtschenkoi*, as well as optimizing nocturnal CO_2_-fixation and malate accumulation, the robustness of the CAM circadian clock, and the associated growth benefits of CAM (Boxall et al., 2017).

In addition to loss-of-function analysis of key CAM components, gain-of-function analysis can also inform the function of key CAM enzymes and transporters. However, there have been few, if any, reports about the direct testing of core C_4_-metabolism cycle genes from CAM species. As a prerequisite for implementing CAM Biodesign, we have selected a set of 13 core C_4_ carboxylation and decarboxylation enzymes and key regulatory proteins based upon their inducible expression patterns in the common ice plant (*Mesembryanthemum crystallinum*), a facultative CAM species (Winter and Holtum, 2014). Each enzyme was overexpressed individually in stably transformed *A. thaliana* plants under the control of a strong constitutive promoter (i.e., CaMV 35S) as C-terminal synthetic green fluorescent protein (sGFP) fusions to determine the subcellular localization of each enzyme. The effects of the overexpression of each enzyme on plant growth were investigated by making detailed measurements of rosette and leaf size and plant biomass production. The relative contributions of each enzyme to stomatal conductance and dawn/dusk titratable acidity (TA) accumulation was also determined. In general, components of the carboxylation module stimulated plant growth and promoted stomatal opening and organic acid accumulation, whereas the decarboxylating malic enzymes stimulated plant growth to a lesser extent and caused stomatal closure and organic acid depletion. These results provide key functional insights into the relative contribution of each of these enzymes and regulators and lay the foundation for introducing CAM into non-CAM species.

## Materials and Methods

### RNA-Sequencing Analysis of Core CAM (C_4_ Enzyme) Genes in Common Ice Plant

Seedlings of the wild-type common ice plant (*M. crystallinum L.*) were grown in a growth chamber (AR-75L2, Percival Scientific Inc., Perry, IA, United States) modified for high light conditions under 12 h/12 h (light, 350 μmol m^-2^ s^-1^/dark) cycles at 26°C/18°C (day/night). Four-week-old plants were subjected to well-watered and water-deficit stressed conditions for 7 days and leaves were collected at each time point 0 (dawn, 6 AM), 4, 8, 12 (dusk, 6 PM), 16, 20, and 24 h (dawn, 6 AM). Total RNA was isolated using the RNeasy Midi Kit with a modified PEG-RNA extraction method that utilized high-molecular weight polyethylene glycol (Gehrig et al., 2000). The cDNA library was sequenced by single-read sequencing using the Illumina HiSeq2000 system. RNA-seq data were assembled using SOAPdenovo-trans v1.03 (Xie et al., 2014) and Trinity release 2013-08-14 (Haas et al., 2013) followed by classification into a non-redundant transcripts set using the EvidentialGene pipeline as a secondary assembler with default parameters as described on the EvidentialGene website^1^. The relative mRNA expression values were then normalized by TMM (trimmed mean of M-values). The averaged FPKM (fragments per kb of exon per million fragments mapped) values of three replicates were then calculated (Robinson and Oshlack, 2010; Trapnell et al., 2010). Examination of the expression values allowed for the identification of specific isogenes encoding key C_4_ enzymes that are involved in the CAM pathway based on their exhibiting increased relative transcript abundance under water-deficit stress conditions compared with well-watered conditions. Only those isogene members within a gene family that showed increased transcript abundance at any time point during the 24-h diel cycle were selected for further analysis (see Figure 2).

### Gene Cloning

To isolate selected core C_4_ enzyme genes from ice plant, the full-length coding sequences of *McBCA2* (beta-carbonic anhydrase 2; iceplant_tr_1475), *McPEPC1* (phosphoenolpyruvate carboxylase 1; iceplant_tr_251007), *McPPCK1* (phosphoenolpyruvate carboxylase kinase 1; iceplant_tr_6059), *McNAD-MDH1* (NAD-malate dehydrogenase 1; iceplant_tr_40435), *McNAD-MDH2* (NAD-malate dehydrogenase 2; iceplant_tr_125991), *McNADP-MDH1* (NADP- malate dehydrogenase 1; iceplant_tr_40439), *McNAD-ME1* [NAD-malic enzyme 1 (alpha subunit); iceplant_tr_249567], *McNAD-ME2* [NAD-malic enzyme 2 (beta-subunit; iceplant_tr_79927)], *McNADP-ME1* (NADP-malic enzyme 1, iceplant_tr_79687), *McNADP-ME2* (NADP-malic enzyme 2, iceplant_tr_11701), *McPPDK* (pyruvate orthophosphate dikinase, iceplant_tr_75614), *McPPDK-RP* (pyruvate orthophosphate dikinase regulatory protein, iceplant_tr_23519), and *McPEPCK* (phosphoenolpyruvate carboxykinase, iceplant_tr_132816) were retrieved from the transcriptome assembly described above. First-strand complementary DNA (cDNA) synthesis from 500 ng of total RNA at indicated time points (unstressed condition; 24 h, drought stress condition; 4, 8, 12, and 24 h) was performed using a SuperScript^®^ III kit (Invitrogen, Carlsbad, CA, United States) according to the manufacturer’s protocol. The core C_4_ enzyme/regulatory genes were amplified with appropriate primer pairs (Supplementary Table S1) using a high-fidelity Pfu Turbo DNA polymerase (Stratagene, La Jolla, CA, United States) from the cDNA mixture. Purified PCR products then were directly introduced into the pENTR^TM^ D-TOPO vector (Invitrogen, Carlsbad, CA, United States) containing attachment L1 and L2 sites for the gateway LR reaction. Next, each gene was cloned into the binary vector ImpGWB405 (CaMV35S::attR1-attR2-sGFP-NOS terminator) as fusion proteins containing C-terminal sGFP (Chiu et al., 1996) by Gateway^TM^ LR Clonase^TM^ II enzyme mix (Nakagawa et al., 2007). For the empty-vector (EV) control, pENTR^TM^ D-TOPO harboring sGFP was cloned into the ImpGWB402 (CaMV35S::attR1-attR2-NOS terminator). Recombinant plasmids were fully sequenced to verify that PCR errors had not occurred.

### *Agrobacterium* Transformation

The recombinant plasmids of the EV control (*CaMV 35S::sGFP*), *CaMV35S::McBCA2-sGFP, CaMV35S::McPEPC1-sGFP, CaMV35S::McPPCK1-sGFP, CaMV35S::McNAD-MDH1-sGFP, CaMV35S::McNAD-MDH2-sGFP, CaMV35S::McNADP-MDH1-sGFP, CaMV35S::McNAD-ME1-sGFP, CaMV35S::McNAD-ME2-sGFP, CaMV35S::McNADP-ME1-sGFP, CaMV35S::McNADP-ME2-sGFP, CaMV35S::McPPDK-sGFP, CaMV35S::McPPDK-RP-sGFP*, and *CaMV35S::McPEPCK-sGFP*, were chemically transformed into the *Agrobacterium tumefaciens* strain GV3101 for floral dipping of *A. thaliana* (ecotype Col-0) (Zhang et al., 2006). T_0_ seeds were harvested and screened on ½ strength MS basal medium containing Gamborg Vitamins (pH = 5.7), 10 g/L sucrose, 50 mg/L kanamycin, and 7 g/L Phytoagar in a Percival Scientific Model CU-32L growth chamber under a 16-h photoperiod for 10 days (light, 135 μmol m^-2^ s^-1^/dark) photoperiod at 23°C/21°C (day/night). T_2_ homozygous seeds were harvested at the same time to minimize differences in seed quality. A total of 5–8 independent T_3_ transformed lines were obtained for each CAM gene. T_3_ plants were subsequently used for subcellular localization and phenotypic characterization. Two independent T_3_ lines were selected for further detailed analysis based on strong sGFP expression intensity.

### Subcellular Localizations of Core C_4_ Enzyme Genes

T_3_ transgenic seedlings of EV control (*CaMV35S::sGFP*), *CaMV35S::McBCA2-sGFP, CaMV35S::McPEPC1-sGFP, CaMV35S::McPPCK1-sGFP, CaMV35S::McNAD-MDH1-sGFP, CaMV35S::McNAD-MDH2-sGFP, CaMV35S::McNADP-MDH1-sGFP, CaMV35S::McNAD-ME1-sGFP, CaMV35S::McNAD-ME2-sGFP, CaMV35S::McNADP-ME1-sGFP, CaMV35S::McNADP-ME2-sGFP, CaMV35S::McPPDK-sGFP, CaMV35S::McPPDK-RP-sGFP*, and *CaMV35S::McPEPCK-sGFP*, were grown on ½ MS basal medium containing Gamborg Vitamins (pH = 5.7), 10 g/L sucrose, and 7 g/L Phytoagar in a Percival Scientific Model CU-32L growth chamber under a 16-h photoperiod for 7 days. Leaf epidermal cells were observed using confocal laser-scanning microscopy (FluoView FV1000, Olympus, Tokyo, Japan). GFP and chloroplast autofluorescence were excited at 488 nm with a laser and emission was collected at 510–560 nm and 680–700 nm, respectively. Subcellular localization predictions were performed using the FUEL-mLoc subcellular localization prediction server at: http://bioinfo.eie.polyu.edu.hk/FUEL-mLoc/citations.html (Wan et al., 2017). This prediction method uses essential GO terms to predict subcellular localizations, allows for multiple subcellular localizations for a protein from many different organisms, and is superior to other prediction programs that use sorting signals and PROSITE patterns.

### Plant Growth Conditions and Biomass Quantification

For phenotypic quantification of vegetative rosette diameter, leaf area, and leaf fresh weight, seeds of each transgenic line were stratified in water at 4°C for 3 days and were directly sown in soil (Sunshine 781, custom blend, 45–50% peat moss, Scotts-Sierra Horticultural Product, Marysville, OH, United States) in 89-mm square plastic pots (Kord, Inc., Toronto, ON, Canada) in a growth chamber (AR-75L2, Percival Scientific Inc., Perry, IA, United States), under a 12-h (light, 150 μmol m^-2^ s^-1^/dark) photoperiod at 23°C/21°C (day/night). Four-week-old rosettes and detached leaves were photographed to measure rosette diameter and the area of fourth true leaves. The fresh weight of the detached leaves was measured directly by gravimetric weighing. Rosette diameter and leaf area were quantified using ImageJ software^2^.

### Leaf Conductance

Four-week-old leaves were used to measure leaf CO_2_ conductance with a SC-1 Leaf Porometer (Decagon Devices, Inc., Pullman, WA, United States) during mid-day hours between 11:00 am and 2:00 pm. Plants were grown in soil in a growth chamber under a 12-h (light, 150 μmol m^-2^ s^-1^/dark) photoperiod at 23°C/21°C (day/night). The leaf CO_2_ conductance was measured on abaxial surface of the fully expanded 4th leaves at locations 3 cm from the base of the lamina.

### Titratable Acidity Assay

Titratable acidity was conducted using transgenic leaf tissues of the 13 C_4_-metabolism cycle genes and the empty-vector control line (Gehrig et al., 2005). Seeds were geminated and plants were grown in soil mixture in a growth chamber under a 12-h photoperiod for 4 weeks. Fully expanded 4th leaves (0.5 g) were collected and immediately ground in liquid nitrogen with a mortar and pestle. Methanol (10 ml, 50% v/v) was added and homogenized samples were boiled at 80°C for 10 min. Water was added to each sample to restore the original volume. Samples were centrifuged at 4000 × *g* for 20 min and then the supernatant was titrated with 100 mM KOH to pH 7.0.

## Results

### Selection of Core C_4_-Metabolism Cycle CAM Genes

**FIGURE 1:**
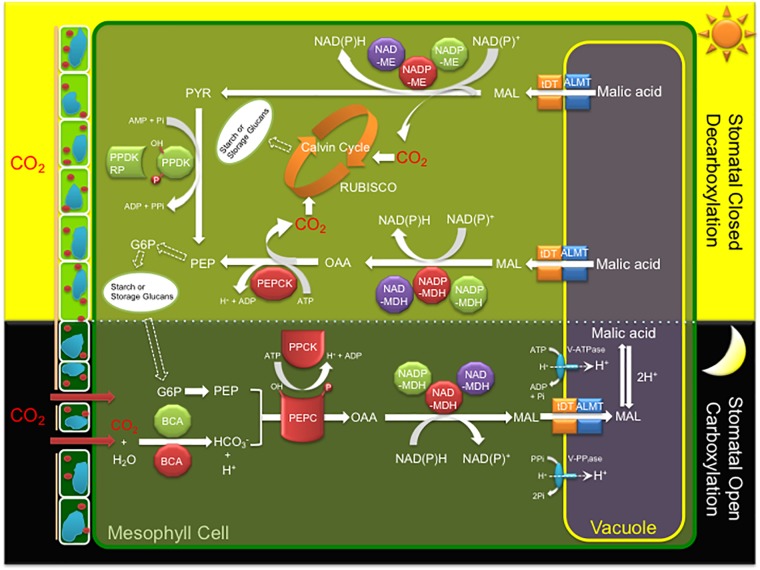
A simplified diagram of the crassulacean acid metabolism (CAM) photosynthetic pathway including key enzymes, regulatory proteins, and transporters of the C_4_ cycle. Key metabolites and transporters: glucose-6-phosphate (*G6P*), malate (MAL), phosphoenolpyruvate (PEP), beta-carbonic anhydrase (BCA), phosphoenolpyruvate carboxylase (*PEPC*), *PEPC* kinase (*PPCK*), NAD(P) malate dehydrogenase (*NAD*(*P*)-*MDH*), ribulose-1,5-bisphosphate carboxylase/oxygenase (RUBISCO), NADP-dependent malic enzyme (*NADP-ME*), pyruvate orthophosphate dikinase (*PPDK*), PPDK regulatory protein (*PPDK-RP*), PEP carboxykinase (*PEPCK*), tonoplast dicarboxylate transporter (*tDT*), aluminum-activated malate transporter (*ALMT*), vacuolar ATPase (*V-ATPase*), and vacuolar pyrophosphatase (*V-PPiase*). Red (cytosol), green (chloroplast), and purple (mitochondria) colors indicate the predicted or experimentally validated subcellular localization of each enzyme or regulatory protein fusion in this study.

CAM is characterized by a core C_4_-metabolism carboxylation module of three major enzymes and a regulatory protein kinase leading to nocturnal CO_2_ uptake and fixation leading to the formation of malate, which is then transported and stored in the vacuole overnight as malic acid (Figure 1). CO_2_ enters the cell through open stomata during the night resulting in less water loss and higher WUE because evapotranspiration rates are lower at night. Beta-carbonic anhydrase (BCA), converts CO_2_ to HCO_3_^-^, which is combined with PEP derived from starch breakdown *via* glycolysis, by PEPC in the cytosol forming oxaloacetate (OAA). PEPC1 is regulated by a minimal, Ca^2+^-independent, Ser/Thr protein kinase, PEPC kinase (PPCK1), which phosphorylates and activates the enzyme relieving its allosteric inhibition by L-malate so that it remains active at night (Taybi et al., 2000). The OAA is then converted to malate by NAD(P)-malate dehydrogenase (MDH) and then transported into the vacuole by either a tonoplast dicarboxylate transporter (tDT) or aluminum-activated malate transporter (ALMT) (Emmerlich et al., 2003; Hurth et al., 2005; Kovermann et al., 2007; Medeiros et al., 2017). Protons for malic acid formation are supplied by the vacuolar ATPase (V-ATPase) and vacuolar pyrophosphatase (V-PPiase) complexes (Kluge et al., 2003; Cosentino et al., 2013). The C_4_ acids accumulated overnight are subsequently decarboxylated during the day to release CO_2_, which is refixed by RUBISCO in the chloroplast, leading to carbohydrate production *via* the Calvin–Benson cycle and gluconeogenesis (Figure 1). Depending on the CAM species, decarboxylation occurs by two pathways. In the first pathway, CO_2_ release occurs by NAD(P)-ME which converts malic acid to pyruvate, which is then regenerated to PEP by PPDK. PPDK activity is modulated by the PPDK-regulatory protein (McPPDK-RP), which activates and deactivates McPPDK by reversible dephosphorylation and phosphorylation, respectively. In the second pathway, malic acid is converted to oxaloacetate by NAD(P)-MDH followed by PEPCK to release CO_2_ and regenerate PEP (Figure 1).

**FIGURE 2:**
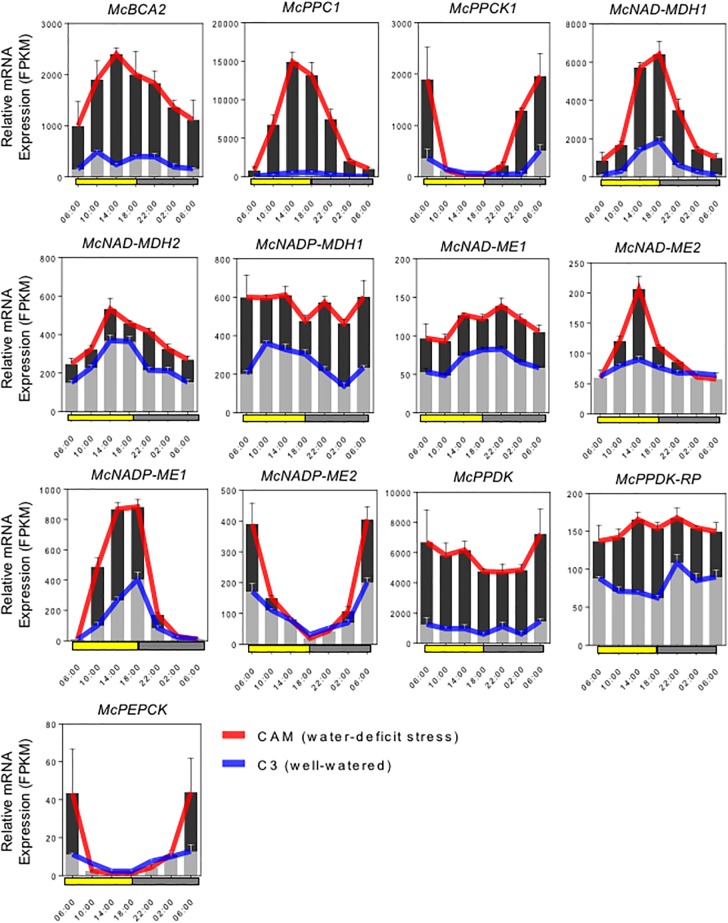
Expression patterns of selected carboxylation/decarboxylation module CAM genes in *M. crystallinum*. To identify specific isogenes, we conducted RNA-sequencing (RNA-seq) in triplicate to define which genes were participating in CAM in the facultative CAM species *M. crystallinum* under well-watered (blue lines) and water-deficit stress (red lines) conditions. The averaged FPKM (fragments per kb of exon per million fragments mapped) values of three biological replicates were calculated from TMM (trimmed mean of *M*-values) normalized RNA-seq data. In summary, six genes of the carboxylation module (*McBCA2*, *McPEPC1*, *McPPCK*, *McNAD-MDH1*, *McNAD-MDH2*, and *McNADP-MDH1)*, seven genes of the decarboxylation module (*McNAD-ME1*, *McNAD-ME2*, *McNADP-ME1*, *McNADP-ME2*, *McPPDK*, *McPPDK-RP*, and *McPEPCK*) were selected for initial CAM biodesign functional testing.

Full-length cDNA clones for the C_4_-metabolism enzymes that comprise the core carboxylation and decarboxylation modules of CAM were identified and isolated by ice plant transcriptome sequencing (Kore-eda et al., 2004; Yim and Cushman, unpublished) and expression profiling (Cushman et al., 2008; Yim and Cushman, unpublished). RNA-seq analysis was conducted in plants performing both C_3_ photosynthesis and CAM induced by water-deficit stress using samples collected in triplicate every 4 h for 24 h. Gene family members for each gene were analyzed for increased steady-state mRNA accumulation following water-deficit stress and candidate genes with likely roles in CAM were selected for further study. Most of these genes also showed pronounced diel (and circadian) changes in mRNA accumulation compared with their expression when plants were unstressed and performing C_3_ photosynthesis. The expression profiles of these candidate genes are summarized in Figure 2.

The C_4_-metabolism carboxylation module genes included *McBCA2*, which showed a substantial increase in relative steady-state transcript abundance with peak expression in the afternoon. The gene encoding *McPEPC1*, a previously characterized CAM-specific isozyme (Cushman et al., 1989), showed very highly increased transcript abundance following CAM induction with peak expression occurring in the afternoon. Relative transcript abundance of the previously characterized *McPPCK11* (Taybi et al., 2000) was also induced with peak expression occurring at dawn. Three distinct genes encoding malate dehydrogenase (MDH) showed increased mRNA abundance following CAM induction. A previously characterized NAD-dependent MDH (*McNAD-MDH1*) (Ocheretina and Scheibe, 1997) showed the greatest transcript abundance increase with peak expression occurring in the afternoon and dusk (Figure 2). A second MDH-NAD encoding gene (*McNAD-MDH2*) also exhibited increased transcript abundance with peak mRNA expression in the late afternoon and evening (Figure 2). A previously characterized CAM-induced NADP-dependent MDH (*McNADP-MDH1*) (Cushman, 1993) showed increased transcript abundance without a clear diel or circadian peak of expression (Figure 2).

The C_4_-metabolism decarboxylation module included genes encoding both NAD- and NADP-malic enzyme (ME), which are responsible for decarboxylating malate to form pyruvate while releasing CO_2_. Two mitochondrial NAD-ME genes encoding the alpha and the beta subunits (*McNAD-ME1* and *2*), respectively, were characterized with peak transcript expression during the early evening and late afternoon, respectively (Figure 2). In addition, a previously characterized CAM-induced NADP-dependent malic enzyme (*McNADP-ME1*) (Cushman, 1992) showed peak expression in the late afternoon and dusk. Interestingly, a second NADP-ME gene (*McNADP-ME2*) was identified also with strongly induced transcript accumulation that peaked at dawn (Figure 2). The pyruvate formed by ME is then phosphorylated to PEP by pyruvate, orthophosphate dikinase (PPDK), which enters the gluconeogenesis pathway. In *M. crystallinum*, a single *McPpdk* gene, described previously (Fißlthaler et al., 1995), showed induced mRNA accumulation with peak expression occurring at dawn (Figure 2). In *M. crystallinum, McPPDK-RP* is encoded by a single gene, which showed increased mRNA abundance in the CAM state with peak expression in late afternoon and early evening (Figure 2). Lastly, a single PEPCK gene (*McPEPCK*) was recovered, which showed very low, but stress-inducible transcript abundance with peak expression at dawn. In summary, these inducible mRNA expression patterns allowed for the unambiguous identification of gene family members encoding enzymes or regulatory proteins with functional roles in CAM.

### Subcellular Localization of C_4_ Metabolism CAM Genes

**FIGURE 3:**
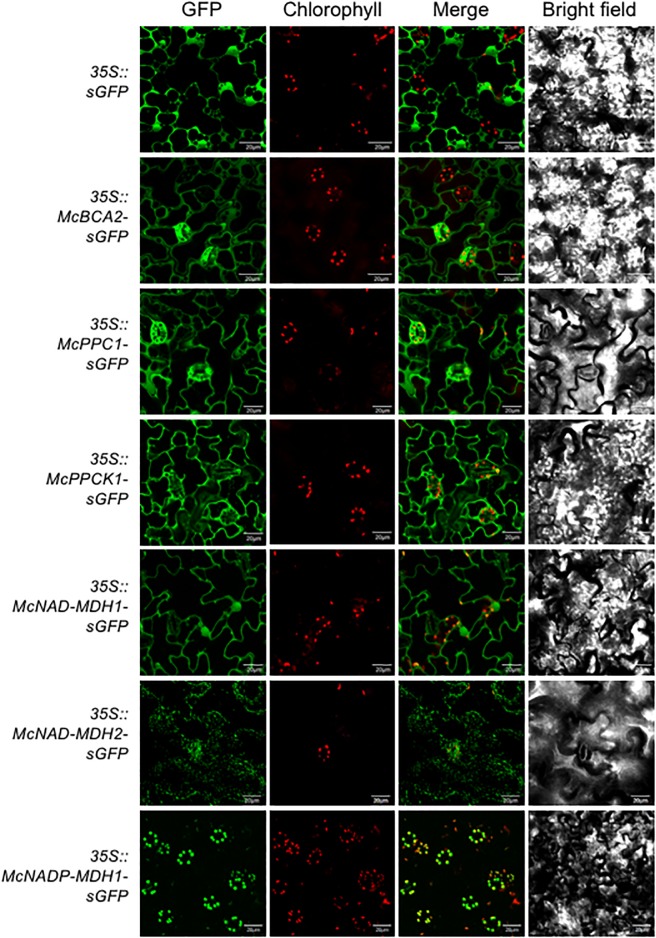
Subcellular localization of selected carboxylation module ice plant CAM genes expressed in *A. thaliana*. To identify subcellular localizations of ice plant CAM genes in *A. thaliana, Agrobacterium* harboring *35S::sGFP* (EV control), *35S::McBCA2-sGFP*, *35S::McPEPC1-sGFP*, *35S::McPPCK1-sGFP*, *35S::McNAD-MDH1-sGFP*, *35S::McNAD-MDH2-sGFP*, or *35S::McNADP-MDH1-sGFP* was transformed into *Arabidopsis* and subcellular localization was determined by confocal microscopy. Bar = 20 μm.

Determining the precise subcellular localization of the enzymes and regulatory proteins essential for CAM is a key prerequisite for validating their predicted subcellular locations and for understanding their precise functional roles. To demonstrate the subcellular localization, each of the core C_4_-metabolism enzymes and regulatory proteins was tagged at their C-terminus with sGFP and expressed under the control of the strong, constitutive CaMV 35S promoter. The C-terminal fusion constructs were then introduced into *A. thaliana* using *Agrobacterium*-mediated transformation. A total of 5–8 independent T_3_ transformed lines were obtained for each CAM gene and two of these lines were selected for further detailed analysis based on strong relative sGFP expression intensity. Leaf epidermal cells were observed using confocal laser-scanning microscopy to visualize sGFP and chloroplast autofluorescence and the images were merged. The *35S::sGFP* fusion construct (empty vector) was used as a control and localized to the cytosol and the nucleus (Figure 3). The initial subcellular location of each protein sequence was predicted using the plant database of the FUEL-mLoc subcellular localization prediction server (Wan et al., 2017).

For the carboxylation module, the McBCA2-sGFP fusion was localized to the cytosol, although it was predicted to localize to the chloroplast. As expected, the McPEPC1-sGFP fusion protein localized in the cytosol (Figure 3). The McPPCK1-sGFP fusion protein also localized to the cytosol to carry out its regulatory role of reversibly phosphorylating PEPC despite a predicted mitochondrial localization. The previously characterized NAD-dependent MDH (McNAD-MDH1) (Ocheretina and Scheibe, 1997) localized to the cytosol (and nucleus), consistent with its predicted cytosolic localization. The second NAD-MDH-encoding gene fusion product (McNAD-MDH2) localized to the mitochondria consistent with its predicted subcellular localization. Lastly, McNADP-MDH1 localized to the chloroplast consistent with its predicted subcellular localization (Cushman, 1993) (Figure 3).

**FIGURE 4:**
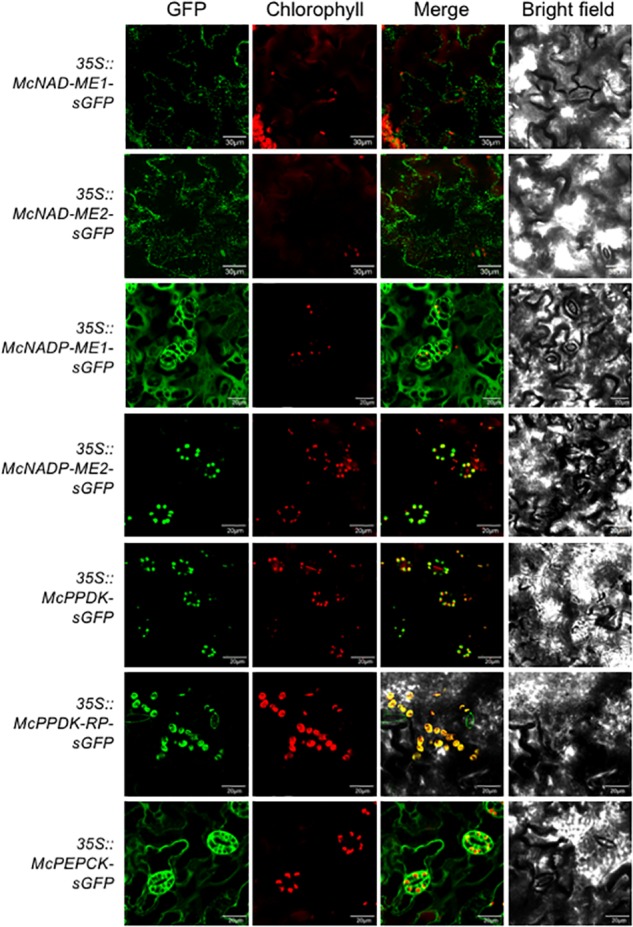
Subcellular localization of selected decarboxylation module ice plant CAM genes expressed in *A. thaliana*. *Agrobacterium* harboring *35S::McNAD-ME1-sGFP*, *35S::McNAD-ME2-sGFP*, *35S::McNADP-ME1-sGFP*, *35S::McNADP-ME2-sGFP*, *35S::McPPDK-sGFP*, *35S::McPPDK-RP-sGFP*, or *35S::McPEPCK-sGFP* was transformed into *A. thaliana* and subcellular localization was determined by confocal microscopy. Bar = 20 μm.

In the decarboxylation module, two NAD-ME genes (*McNAD-ME1* and *McNAD-ME2*) encoding the alpha and the beta subunits, respectively, localized to the mitochondria consistent with their predicted subcellular locations (Figure 4). McNADP-ME1 localized to the cytosol, consistent with its predicted cytosolic localization. McNADP-ME2, predicted to localize to the chloroplast, was confirmed to localize to the chloroplast (Figure 4). McPPDK was shown to be localized to the chloroplast as predicted (Figure 4). As expected, McPPDK-RP was also localized to the chloroplast, consistent with a predicted localization to chloroplast. Although a minor component of the decarboxylation pathway in *M. crystallinum*, PEPCK was localized to the cytosol consistent with its predicted subcellular localization (Figure 4). These results demonstrate the importance of performing empirical subcellular localization testing as subcellular localization prediction programs are not always accurate as was the case for *McBCA2* and *McPPCK1*.

### Phenotypic Analysis of C_4_ Metabolism Gene Overexpression Lines

**FIGURE 5:**
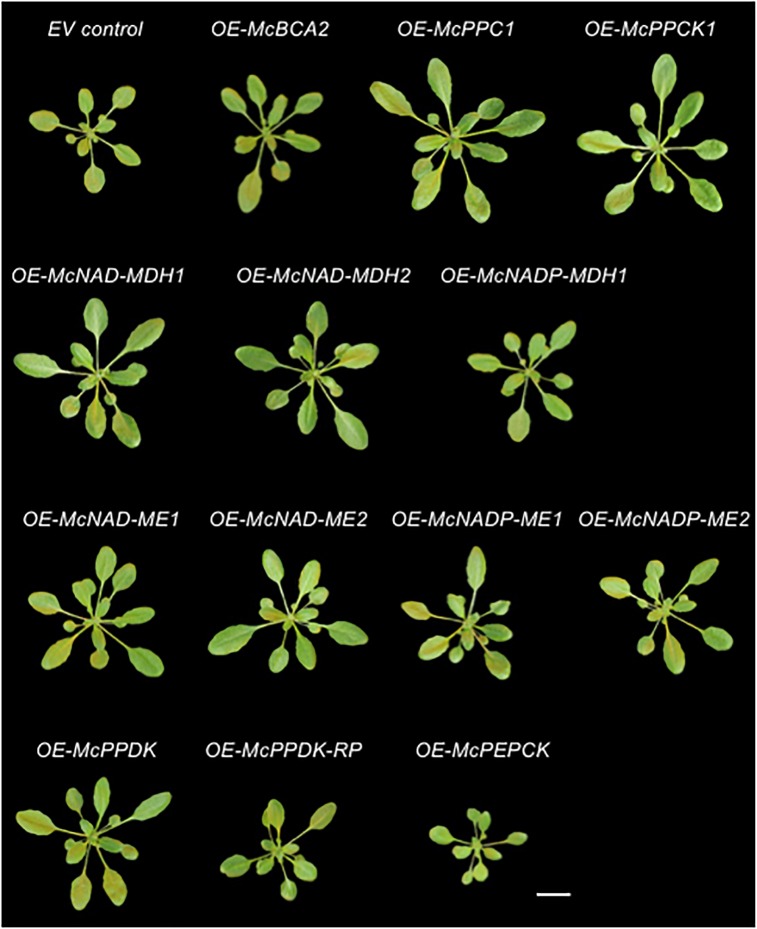
Overexpression of individual carboxylation/decarboxylation module ice plant CAM genes alters overall plant size in *A. thaliana*. Representative images of 4-week-old *35S::sGFP* Empty Vector (EV) control, *35S::McBCA2-sGFP*, *35S::McPEPC1-sGFP*, *35S::McPPCK1-sGFP*, *35S::McNAD-MDH1-sGFP*, *35S::McNAD-MDH2-sGFP*, *35S::McNADP-MDH-sGFP*, *35S::McNAD-ME1-sGFP*, *35S::McNAD-ME2-sGFP*, *35S::McNADP-ME1-sGFP*, *35S::McNADP-ME2-sGFP*, *35S::McPPDK-sGFP*, *35S::McPPDK-RP-sGFP*, or *35S::McPEPCK-sGFP* transgenic plants. Bar = 2 cm.

**FIGURE 6:**
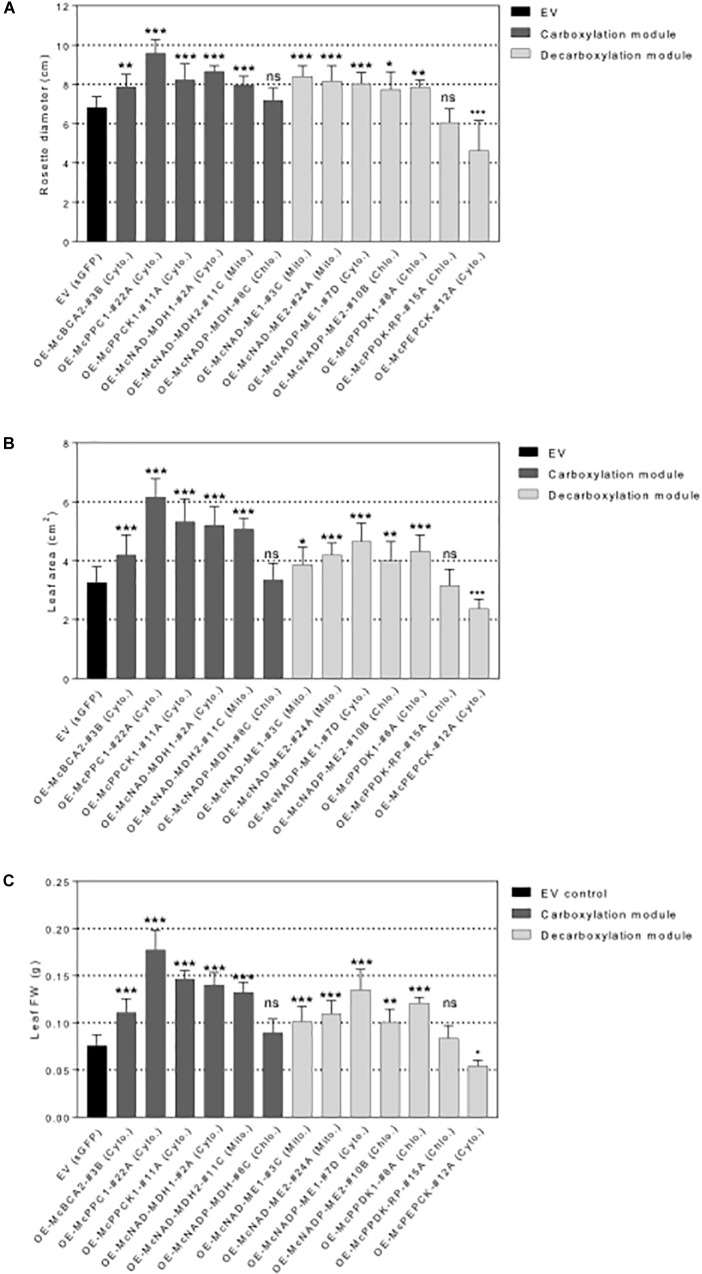
Overexpression of individual carboxylation/decarboxylation module ice plant CAM genes alters plant biomass in *A. thaliana*. T_3_ homozygous seeds of *35S::sGFP* Empty Vector (EV) control, *35S::McBCA2-sGFP*, *35S::McPEPC1-sGFP*, *35S::McPPCK1-sGFP*, *35S::McNAD-MDH1-sGFP*, *35S::McNAD-MDH2-sGFP*, *35S::McNADP-MDH1-sGFP*, *35S::McNAD-ME1-sGFP*, *35S::McNAD-ME2-sGFP*, *35S::McNADP-ME1-sGFP*, *35S::McNADP-ME2-sGFP*, *35S::McPPDK-sGFP*, *35S::McPPDK-RP-sGFP*, and *35S::McPEPCK-sGFP* were germinated and grown in soil mix under a 12-h photoperiod. 4-week-old plants were used to analyze overall plant biomass. **(A)** Quantification of rosette diameter (*n* = 10). **(B)** Quantification of leaf area (*n* = 20). **(C)** Quantification of leaf fresh weight (*n* = 20). Values represent means ± SD, ns, non-significant, ^∗^*p* < 0.05, ^∗∗^*p* < 0.01, and ^∗∗∗^*p* < 0.001, One-way ANOVA with Dunnett’s multiple comparison test.

In addition to confirming the subcellular localization of each CAM enzyme and cognate regulatory proteins, the stably transformed C_4_-metabolism gene-sGFP fusion lines allowed for the investigation of possible phenotypic effects of overexpressing each of these genes individually under the control of the strong, constitutive CaMV 35S promoter as a way of understanding their relative contributions to an engineered CAM phenotype. A total of 5–8 independent T_3_ transgenic lines were obtained for each CAM gene and two independent lines were selected for further analysis based on strong relative sGFP expression (see Supplementary Figure S1). The phenotypes of representative homozygous, T_3_ generation plants for the empty vector (EV) control line *35S::sGFP* expressing sGFP alone and each of the CAM-gene-sGFP fusion lines were imaged after 4 weeks of growth on soil (Figure 5). The rosette and leaf morphology of each of the C_4_-metabolism gene overexpression lines appeared normal except for apparent variations in size. No lines exhibited any notable leaf necrosis or early senescence. Next, the rosette diameters and leaf areas of the 4th leaf of each line was quantified using ImageJ software (Supplementary Figure S1 and Figure 6A,B). The fresh weights of all rosette leaves were also measured (Supplementary Figure S1 and Figure 6C).

All C_4_-metabolism gene-sGFP fusion lines expressing carboxylation-module protein fusions showed significantly larger rosette diameters, 4th leaf areas, and total leaf fresh weights than the EV control line except for the *35S::McNADP-MDH1-sGFP* expressing line, which showed no significant increases in these measurements (Figure 6). The 3*5S::McPEPC1-sGFP* fusion line showed 1.4-fold increase in rosette diameter, a 1.3-fold increase in 4th leaf area, and a 2.3-fold increase in total leaf fresh weight compared to the EV control line.

All C_4_-metabolism gene-sGFP fusion lines expressing decarboxylation-module protein fusions showed significantly larger rosette diameters, 4th leaf areas, and total leaf fresh weights than the EV control line except for the *35S::McPPDK-RP-sGFP* line, which showed no significant increase, and the *35S::McPEPCK-sGFP* line, which showed a significant decrease in these phenotypic parameters (Figure 6). The *35S::McPEPCK-sGFP* fusion line showed 1.3-fold decrease in rosette diameter, 4th leaf area, and total leaf fresh weight compared to EV control (sGFP) line. These results demonstrated that all of the decarboxylation enzymes (and *McPPCK1*) tested had a positive impact on leaf growth in *A. thaliana*.

### CAM-Like Phenotypes of C_4_ Metabolism Gene Overexpression Lines

**FIGURE 7:**
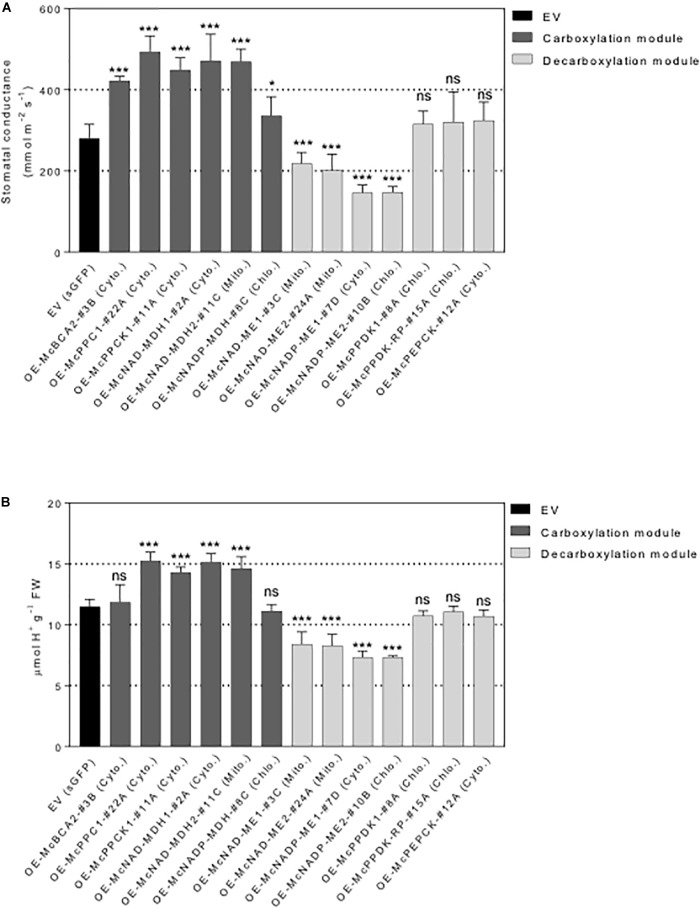
Overexpression of individual carboxylation/decarboxylation module ice plant CAM gene alters leaf stomatal conductance and malate content in *A. thaliana*. T_3_ homozygous seeds of *35S::sGFP* Empty Vector (EV) control, *35S::McBCA2-sGFP*, *35S::McPEPC1-sGFP*, *35S::McPPCK1-sGFP*, *35S::McNAD-MDH1-sGFP*, *35S::McNAD-MDH2-sGFP*, *35S::McNADP-MDH1-sGFP*, *35S::McNAD-ME1-sGFP*, *35S::McNAD-ME2-sGFP*, *35S::McNADP-ME1-sGFP*, *35S::McNADP-ME2-sGFP*, *35S::McPPDK-sGFP*, *35S::McPPDK-RP-sGFP*, and *35S::McPEPCK-sGFP* were germinated and grown in soil mix under a 12-h photoperiod. Four-week-old leaf was used. **(A)** Quantification of stomatal conductance (*n* = 10). **(B)** Quantification of malate content (*n* = 10). Values represent means ± SD, ns, non-significant, ^∗^*p* < 0.05 and ^∗∗∗^*p* < 0.001, One-way ANOVA with Dunnett’s multiple comparison test.

To further investigate the underlying mechanistic basis of the changes in growth and biomass accumulation observed in the overexpression lines, the stomatal behavior of each representative control EV lines and C_4_-metabolism gene-sGFP fusion lines was determined. A significant increase in stomatal conductance was observed for all the carboxylation module overexpression lines, including the *35S::McNADP-MDH1-sGFP* expressing line, relative to the control EV line, although the value for this line was less significant than the other carboxylation-module lines (Figure 7A). The *35S::McPEPC1-sGFP* fusion line showed 1.7-fold increase in stomatal conductance compared to the EV control line. These results suggest that the activity of the carboxylation enzymes and McPPCK1 was promoting stomatal opening.

In contrast, four of the decarboxylation module C_4_-metabolism-gene ME overexpression lines showed significant decreases in stomatal conductance. The *35S::McNAD-ME1-sGFP* and *35S::McNAD-ME2-sGFP* fusion lines each showed a 1.3-fold decrease in stomatal conductance compared to the EV control line. The *35S::McNADP-ME1-sGFP* and *35S::McNADP-ME2-sGFP* fusion lines each showed a 1.5-fold decrease in stomatal conductance compared to the EV control line. These results suggest that the activity of these malate decarboxylation enzymes was promoting stomatal closure. In contrast, the overexpression of McPPDK1 and McPPDK-RP in *A. thaliana* failed to cause significant changes in stomatal conductance presumably because these enzymes are not involved directly in the release of CO_2_ or malate decarboxylation within the leaf. Lastly, the overexpression of McPEPCK, which involves the release of CO_2_ during the conversion of OAA to PEP, did not result in a change in stomatal conductance (Figure 7A).

In order to confirm that the observed changes in stomatal conductance were linked with changes in malate formation or degradation, each of the carboxylation/decarboxylation module C_4_-metabolism gene-sGFP fusion overexpression lines were evaluated by TA analysis, which is an indirect measure of organic acid (mainly malate in ice plant) accumulation (Herppich et al., 1995). The *35S::McBCA2-sGFP* overexpression line did not show a significant change in TA (Figure 7B). However, the *35S::McPEPC1-sGFP*, *35S::McPPCK1:sGFP*, and the *35S::McNAD-MDH1-sGFP* and *35S::McNAD-MDH2-sGFP*, expressing lines all showed significant 1.2- to 1.3-fold increases in organic acid formation. These results suggest that each of these enzymes, or regulatory protein kinase in the case of McPPCK1, can promote malate formation in *A. thaliana* consistent with the observed increases in stomatal opening. The 35S::*McNADP-MDH-sGFP* overexpression line did not exhibit a significant change in TA.

In contrast, all four of the decarboxylation module *35S::McNAD(P)-ME-sGFP* overexpression lines showed significant ∼1.3-fold decreases in organic acid accumulation, presumably because of malate decarboxylation, which corresponded with reductions in stomatal conductance (Figure 7B). In contrast, the *35S::McPPDK-sGFP* and *35S::McPPDK-RP-sGFP* lines failed to result in significant changes in organic acid accumulation, presumably because these enzymes are not directly involved in malate production. Similarly, the *35S::McPEPCK-sGFP* line, which is also not directly involved in malate biosynthesis, did not exhibit a significant change in TA (Figure 7B). Overall, the observed trends in TA accumulation patterns were consistent with the observed changes in stomatal conductance and indicate a strong correlation between stomatal conductance and organic acid accumulation.

## Discussion

The introduction of the water-saving CAM pathway into conventional C_3_- (or C_4_-) photosynthesis crops represents a potentially useful strategy to improve WUE while maintaining high productivity (Borland et al., 2014, 2015; Yang et al., 2015). One of the major prerequisites of CAM Biodesign is the ability to define the functionality and relative contribution of each component of the system in a non-CAM genetic background. In this report, the mRNA expression, subcellular localization, and phenotypic effects of overexpressing 13 enzymes and regulatory proteins of the core carboxylation/decarboxylation modules of C_4_-metabolism cycle of CAM were analyzed. Understanding the relative contribution or lack thereof for each individual component of this core C_4_-metabolism is a critical first step in laying the foundation for CAM Biodesign.

### Identification of CAM Enzymes

As a facultative CAM species, *M. crystallinum* allows for the facile identification of C_4_-metabolism genes of the CAM cycle based on their inducible expression patterns following salinity or water-deficit stress (Cushman et al., 2008; Yim and Cushman, unpublished). The C_4_-metabolism carboxylation module genes included a strongly induced *McBCA2*, which was suggested previously to play a key role in the primary nocturnal CO_2_ fixation of CAM (Cushman et al., 2008). This gene encodes an ortholog to the *A. thaliana AtBCA2* gene (At5g14740), which is abundantly expressed, localized to the cytosol, and is important for optimal plant growth at low CO_2_ concentrations (Fabre et al., 2007; DiMario et al., 2016). The inducible, CAM-specific *McPEPC1* gene, also showed very high transcript expression following CAM induction consistent with previous observations (Cushman et al., 1989; Cushman et al., 2008). The CAM-induced transcript abundance pattern of *McPPCK1* was also consistent with previous observations (Taybi et al., 2000). Among the MDH genes, the mRNA abundance patterns of the *McNAD-MDH1*, predicted to encode a cytosolic enzyme, were also consistent with previous observations (Ocheretina and Scheibe, 1997) with peak expression occurring in the afternoon and dusk (Figure 2). *McNAD-MDH2* exhibited increased transcript abundance with peak mRNA expression in the late afternoon and evening. These expression patterns were consistent with NAD-MDH activity being the predominant enzyme activity detected in CAM-performing *M. crystallinum* (Holtum and Winter, 1982; Winter et al., 1982a). In addition, a CAM-induced gene encoding *McNADP-MDH1* (Cushman, 1993) was implicated previously as playing roles in CAM based upon enzyme activity increases in the non-cytosolic leaf fraction following CAM induction (Holtum and Winter, 1982; Winter et al., 1982a).

The C_4_-metabolism decarboxylation module in *M. crystallinum*, includes mitochondrial localized NAD-ME as the predominant enzyme activity in CAM-performing leaves (Holtum and Winter, 1982; Winter et al., 1982a, 1986). Consistent with this observation, two mitochondrial NAD-ME genes encoding the alpha and the beta subunits (*McNAD-ME1* and *McNAD-ME2*), respectively, were characterized with inducible transcript expression (Figure 2). In addition, NADP-ME activity was also reported in ice plant (Holtum and Winter, 1982). The previously characterized gene (*McNADP-ME1*) encoding a cytosolic form of the enzyme (Cushman, 1992; Cushman et al., 2008), showed strong transcript accumulation following CAM induction consistent with a role in daytime malate decarboxylation. A second NADP-ME gene (*McNADP-ME2*) encoding a chloroplastic form of the enzyme was newly identified with strongly induced transcript accumulation (Figure 2). The pyruvate formed by ME is then phosphorylated to PEP by PPDK, which enters the gluconeogenesis pathway. In *M. crystallinum*, a single *McPPDK* gene, described previously (Fißlthaler et al., 1995), showed a pronounced increase in transcript abundance in CAM (Figure 2). PPDK is activated and deactivated by reversible dephosphorylation and phosphorylation, respectively, catalyzed by PPDK-RP (Astley et al., 2011). In *M. crystallinum*, PPDK-RP is encoded by a single gene, which showed increased mRNA abundance in the CAM state (Figure 2). Lastly, although PEPCK activity, which converts OAA to PEP, was undetectable in ice plant extracts (Holtum and Winter, 1982), a stress-inducible transcript encoding a single gene was recovered, suggesting it might play a role in daytime decarboxylation of OAA. However, based upon the relative transcript abundance values observed for *McPPDK* compared with those observed for *McPEPCK* (Figure 2), the MDH-PPDK decarboxylation pathway is clearly the predominant pathway in the common ice plant.

### Subcellular Localization of CAM Enzymes

The precise subcellular localization of the enzymes and regulatory proteins essential for CAM was validated by fusing the C-terminus of each protein-coding region to sGFP and expressing each one under the control of the strong, constitutive CaMV 35S promoter. Such validation is necessary because subcellular localization prediction algorithms are often inaccurate. For example, the subcellular locations predicted for two (*McBCA2* and *McPPCK1*) of the 13 proteins tested in this study was incorrect when using the FUEL-mLoc subcellular localization prediction server (Wan et al., 2017). Of course, there is a remote possibility that the observed subcellular localization for these protein fusions was incorrect due to the C-terminal sGFP fusion. However, this is unlikely and the incorrect predicted localization results might simply reflect a limitation of the FUEL-mLoc subcellular localization prediction software itself as these proteins are expected to be localized to the cytosol consistent with their known roles.

Within the carboxylation module, the observed cytosolic localization of the McBCA2-sGFP fusion to the cytosol was consistent with its expected role in providing HCO_3_^-^ substrate for PEPC, which was also localized to the cytosol (Winter et al., 1982a). Other BCA activities in *M. crystallinum* leaves are known to localize in the chloroplast as BCA is distributed between the chloroplast:cytosol in a 80:20 ratio (Tsuzuki et al., 1982). These BCA activities might also participate in the recycling of nocturnal photorespiratory CO_2_ in CAM, which helps to maintain carbon balance and prevent photoinhibition (Herrera, 2009, 2013). As expected, McPEPC1-sGFP fusion localized in the cytosol (Figure 3), which is consistent with previous observations from subcellular fractionation studies (Tsuzuki et al., 1982; Winter et al., 1982a). The localization of the McPPCK1-sGFP fusion to the cytosol was consistent with its regulatory role of reversibly phosphorylating PEPC to reduce its sensitivity to allosteric L-malate inhibition at night (Taybi et al., 2000). The previously characterized McNAD-MDH1 (Ocheretina and Scheibe, 1997) localized to the cytosol (and nucleus), which was consistent with subcellular fractionation studies in *M. crystallinum* (Holtum and Winter, 1982; Winter et al., 1982a). The localization of McNAD-MDH2 to the mitochondria and of McNADP-MDH1 to the chloroplast confirmed their predicted subcellular localizations (Figure 3).

Within the decarboxylation module, the mitochondrial localization of McNAD-ME1 and McNAD-ME2, which encode the alpha and the beta subunits, respectively (Figure 4), was consistent with increased NAD-ME activity in the non-cytosolic fraction in CAM-performing *M. crystallinum* (Winter et al., 1982a, 1986). McNADP-ME1 was confirmed to localize to the cytosol (Figure 4) as this enzyme was shown to be the predominant decarboxylating enzyme activity in CAM-performing *M. crystallinum* (Winter et al., 1982a). In contrast, McNADP-ME2 was confirmed to localize to the chloroplast (Figure 4) consistent with increased NADP-ME activity in the non-cytosolic fraction in CAM-performing *M. crystallinum* (Winter et al., 1982a). The localization of McPPDK solely within chloroplasts was consistent with the localization of its activity to the chloroplast fraction (Winter et al., 1982a) (Figure 4). The subcellular localization of McPPDK to the plastid was also consistent with immunolocalization studies that showed that McPPDK was present only in *M. crystallinum* chloroplasts (Kondo et al., 1998). However, depending on the genus of CAM species, PPDK can localize to either the plastid, both the cytosol and the chloroplast, or the cytosol (Kondo et al., 1998, 2000). In those species with plastid-localized PPDK, NADP-ME activity tended to be greater than NAD-ME (as in ice plant), whereas species with cytosolic-localized PPDK tended to have NAD-ME activities that were greater or equivalent to NADP-ME activity levels (Kondo et al., 2000). The observation that *McPPDK* localizes solely within chloroplasts also differs from observations made in *A. thaliana* where the *AtPpdk1* gene (AT4G15530) can encode a protein that is localized to either the chloroplast or the cytosol depending upon the production of alternative transcripts arising from two different promoters (Parsley and Hibberd, 2006). Investigation into the possibility of such alternative splicing should be undertaken in CAM species to confirm that similar mechanisms account for the various PPDK localizations observed among various CAM species. As expected, the *McPPDK-RP* gene product was also localized to the chloroplast where it is expected to activate and deactivate *McPPDK* by reversible dephosphorylation and phosphorylation, respectively (Figure 4). Although PEPCK is a minor component of the decarboxylation pathway in *M. crystallinum*, it’s localization to the cytosol was consistent with its predicted subcellular localization. PEPCK expression was obvious within stomatal guard cells. In *A. thaliana* PEPCK is expressed in guard cells and is implicated in promoting dark-induced stomatal closure presumably by way of gluconeogenesis to convert vacuolar malate to starch (or sucrose) (Penfield et al., 2012). The localization of the McPEPCK-sGFP fusion to the cytosol with apparent localization within stomatal guard cells suggests that McPEPCK might play a similar role in promoting stomatal closure in *M. crystallinum* (Figure 4).

### Morphometric Analysis of CAM Enzyme Overexpression Lines

The relative contribution of each C_4_ metabolism enzyme or regulatory protein to plant growth was evaluated by assessing rosette size, 4th leaf size, and total rosette fresh weight. The overexpression of most of the carboxylation-module enzymes resulted in significantly larger plants compared with the EV control line, except for the *35S::McNADP-MDH1-sGFP* expressing line, which showed no significant increase in plant or leaf size (Figure 5, 6). The improved growth of four of the six lines was also well correlated with organic acid accumulation (Figure 7B). Reduced accumulation of malate and fumarate (and starch) has been shown to be well correlated with reduced growth in *tDT* knockout mutants of *A. thaliana* (Medeiros et al., 2017). This reduced growth was suggested to occur because of carbon-starvation, particularly apparent when plants were grown under short-day (8 h) conditions, due to the accelerated usage of cytosolic carboxylic acids as an energy source.

The growth stimulation observed is similar in some instances to the effects of overexpression of several C_4_ metabolism enzymes from other species. The first step of the core C_4_ -metabolism cycle is catalyzed by BCA. While loss of either AtBCA2 or AtBCA4, the two most abundant cytosolic versions of this enzyme in *A. thaliana*, had no effect on plant growth. Loss of both AtBCA2/AtBCA4 results in a significant reduction in plant growth at low [CO_2_], but not at high [CO_2_] (DiMario et al., 2016). However, overexpression of AtBCA2 and AtBCA4 resulted in a slightly larger rosette size in *A. thaliana*. These results suggested that these highly abundant BCA enzymes are essential for the proper function of PEPC in the production of amino acids and possibly other anaplerotic metabolic pathways in *A. thaliana* that might have a direct impact on plant growth.

The overexpression of PEPC had the largest growth stimulation effect among all the carboxylation enzymes. The growth stimulation effects of overexpressing various types of PEPC in transgenic plants have been reported (Häusler et al., 2002; Raines, 2006). Early reports of overexpression of C_4_-PEPC from maize in transgenic tobacco resulted in a reduction in plant growth with a reduction in photosynthesis under elevated O_2_ conditions (Kogami et al., 1994). Retarded growth rates were also observed for transgenic potato grown in axenic culture expressing a bacterial form of PEPC isolated from *Corynebacterium glutamicum* compared with wild-type control (Gehlen et al., 1996). Similiar retarded growth phenotypes were observed for transgenic potato lines overexpressing a C_4_-photosynthesis-like form of PEPC with a reduced Km for PEP, increased *I*_50_ for malate, and an increased substrate affinity, under the control of the CaMV 35S promoter (Rademacher et al., 2002). However, the basis of this growth inhibition was not understood and was not observed in lines expressing a C_3_-photosynthesis-like PEPC. In contrast, overexpression of maize PEPC in transgenic rice was reported to increase photosynthetic capacity, biomass accumulation, and grain yield (Ku et al., 2000; Gu et al., 2013). The increased photosynthetic capacity was suggested to be due, in part, to increased stomatal conductance and higher internal CO_2_ concentrations. Such increased stomatal conductance was like that observed for the *35S::McPEPC1-sGFP* expressing lines (Figure 7A). Interestingly, like McPEPC, the overexpression of McPPCK resulted in a growth stimulation relative to the empty-vector control line (Figure 5, 6), as well as increased malate production and stomatal conductance (Figure 7), suggesting that it can act upon the endogenous *A. thaliana* PEPC and stimulate or extend its activity over the diel cycle. This is highly likely given that this protein kinase can phosphorylate ice plant PEPC as well as other recombinant PEPC enzymes from C_4_ plant species as substrates (Taybi et al., 2000). However, additional experimentation is needed to confirm this suggestion.

Both the cytosolic *35S::McNAD-MDH1-sGFP* and mito-chondrial *35S::McNAD-MDH2-sGFP* overexpressing lines expressing the chloroplast-localized NADP-dependent form of MDH also showed significant growth stimulation (Figure 5, 6); however, the growth effects were less than those arising from PEPC overexpression. In contrast, antisense silencing of a mitochondrial NAD-MDH in transgenic tomato resulted in an increase in growth and CO_2_ assimilation rates and aerial plant dry matter (Nunes-Nesi et al., 2005). The mechanism of such growth stimulation was unclear from this report, but was thought to be linked to an ascorbate-mediated stimulation of photosynthesis in this instance. Loss-of-function double T-DNA insertion mutants lacking both mitochondrial NAD-MDH1 and 2 showed significant growth defects with low net CO_2_ assimilation rates compared with wild-type plants linked to elevated leaf respiration (Tomaz et al., 2010). Complementation from overexpression of the corresponding mMDH1 enzyme under the control of the CaMV 35S promoter resulted in a restoration of the wild-type phenotype, but growth stimulation, while apparent, was not quantified in this study. In contrast to the NAD-MDH enzymes, the *35S::McNADP-MDH1-sGFP* overexpressing lines expressing chloroplast-localized NADP-MDH showed no significant growth stimulation (Figure 6). This enzyme, which is the key enzyme of the malate valve, which consumes NADPH during the conversion of OAA to malate facilitating the regeneration of the electron acceptor NADP^+^ in the chloroplast to maintain redox homeostasis (Scheibe, 2004), did not result in increased malate accumulation as confirmed by the lack of increased TA in this line (Figure 7). However, increased leaf area and leaf and shoot biomass production was reported from the overexpression of a chloroplast-localized NADP-MDH from pea when expressed in transgenic tobacco within a defined developmental window (Faske et al., 1997). The increased growth was correlated with increased malate formation and export and an optimal ATP/NADPH ratio within chloroplasts. Overexpression of a chloroplast-localized NADP-MDH from pea in transgenic potato plants showed increased malate accumulation, but a growth analysis of these plants was not performed (Backhausen et al., 1998). Knockout lines of the chloroplast-localized NADP-MDH ortholog in *A. thaliana* showed no effect on plant growth in adult plants, but young plants lacking this enzyme displayed enhanced growth early in development (Hebbelmann et al., 2011).

The overexpression of both McNAD-ME and McNADP-ME enzymes resulted in significant increased plant size and biomass accumulation (Figure 5, 6) and the plants were healthy in appearance. The growth stimulation observed in these lines might arise from the intracellular CO_2_ release, thereby reducing photorespiration, which could assist in promoting growth. These enzymes also result in the production of PYR, which readily enters the TCA cycle and could result in an anaplerotic growth stimulation in these plants. Knock-out lines of *AtNAD-ME1*, *AtNAD-ME2* and double knockout lines of both enzymes showed no significant reduction in plant dry biomass or rosette size suggesting that these enzymes are not essential for normal autotrophic development (Tronconi et al., 2008). However, metabolic profiling of rosette leaves revealed that the *nad-me* loss of function mutants accumulated excess nocturnal malate, which was diverted from TCA cycle intermediates into amino acids.

The *McNADP-ME* overexpression line results were consistent with the reduced size of knock-out mutants of *AtNADP-ME2*, which is orthologous to *McNADP-ME2* (Li et al., 2013). However, these results differed substantially from studies that showed that overexpression of a maize chloroplastic NADP-ME in transgenic rice showed aberrant chloroplasts with agranal thylakoids (Takeuchi et al., 2000) and impaired auxotrophic growth (Tsuchida et al., 2001). Overexpression of a maize C_4_ NADP-ME enzyme in *A. thaliana* revealed a reduction in rosette size and biomass as a consequence of thinner leaves with lower chlorophyll content and reduced CO_2_ assimilation rates when plants were grown under short-day conditions (Zell et al., 2010). Overexpression of *AtNADP-ME2* in *A. thaliana* resulted in reduced rosette size, root length, delayed flowering, increased sensitivity to osmotic stress, and increased starch accumulation (Badia et al., 2015).

The *35S::McPPDK-sGFP* expressing lines showed growth stimulation, which has been observed in other selected studies. The growth stimulation observed in these lines might arise from the production of PEP, which is used by PEPC to promote anaplerotic functions in these plants. Alternatively, PEP can be readily converted to PYR with the release of ATP, which readily enters the TCA cycle and likely stimulating growth in these plants. In earlier studies, the expression of a functional maize C_4_ PPDK in transgenic *A. thaliana* and potato was achieved, but expression levels were likely not high enough to alter carbon metabolism or photosynthetic parameters (Ishimaru et al., 1997; Ishimaru et al., 1998). In contrast, McPPDK overexpression in transgenic tobacco resulted in greater seed yield than wild-type controls (Sheriff et al., 1998). In this case, PPDK was thought to increase PEP supply thereby stimulating the anaplerotic action of PEPC through the capture of respiratory CO_2_ in developing seeds. These results also resembled the overexpression of a maize PPDK in transgenic rice, which resulted in increased photosynthetic capacity, biomass accumulation, and grain yield (Ku et al., 2000; Gu et al., 2013). The increased photosynthetic capacity was thought to be the result of increased stomatal conductance and higher internal CO_2_ concentrations. However, for the *35S::McPPDK-sGFP* expressing lines, no significant increase in stomatal conductance relative to control lines was observed (Figure 7A). Although understanding the basis of the observed growth stimulation by McPPDK overexpression requires additional experimentation, this enzyme likely increases anaplerotic flux through PEPC. In contrast, the overexpression of McPPDK-RP showed that the overexpression of this regulatory protein by itself had no significant influence on plant growth, presumably because without its native target enzyme, it cannot activate or deactivate McPPDK1 by reversible dephosphorylation and phosphorylation, respectively, and thereby influence plant growth. Alternatively, there was insufficient expression of the *A. thaliana* PPDK substrate to alter carbon flux to a notable degree.

The increased McPEPCK-sGFP expression significantly reduced plant and leaf size and leaf biomass production. The overexpression of PEPCK from the PCK-type C_4_ species *Urochloa panicoides* in transgenic rice plants, resulted in increased carbon flow through a C_4_-like pathway and shorter culm and panicle lengths than wild-type controls (Suzuki et al., 2000). The exact basis for smaller plants is unclear, but excess amounts of this enzyme might alter carbon flux by reducing OAA accumulation thereby limiting flux through the TCA cycle and reducing growth. However, detailed metabolic profiling will have to be performed to confirm such metabolite alterations. The overexpression of PEPCK from the bacterium *Sinorhizobium meliloti* in chloroplasts of transgenic tobacco plants had little effect on photosynthetic parameters likely due to the low expression levels achieved (Häusler et al., 2001).

### CAM-Like Phenotypes of CAM Enzyme Overexpression Lines

Increased stomatal conductance was observed for all the carboxylation module overexpression lines (Figure 7). This result suggested that each of these enzymes, and the regulatory protein kinase in the case of McPPCK1, can promote stomatal opening. Reduced stomatal conductance was observed for the McNAD-ME and McNADP-ME overexpression lines of the decarboxylation module. Furthermore, stomatal opening and closing was strongly correlated with TA (malate) concentrations. However, the exact mechanisms for the observed changes in stomatal conductance remain unclear. In one scenario, lowering internal leaf CO_2_ concentrations by the action of these carboxylation module enzymes (or regulatory protein kinase in the case of McPPCK1) to form malate might promote stomatal opening through the CO_2_-sensing pathway (Engineer et al., 2016). Direct measurements of intracellular CO_2_ concentrations are needed to confirm this scenario. In a second scenario, the increased accumulation of malate as indicated by increased TA measurements in these lines, had a direct effect on stomatal guard cells to promote stomatal opening. Malate is well established to stimulate malate-activated vacuolar chloride channels, such as AtALMT9 in *A. thaliana* guard cells, leading to stomatal opening (De Angeli et al., 2013). Small increases in malate accumulation in the cytosol are enough to stimulate stomatal opening via AtALMT9, which mediates the influx of Cl- into the vacuole of guard cells. However, direct measurement of malate content is necessary to validate this hypothesis as other organic acids, such as citrate, can also contribute to TA measurements. Other K^+^ and NO_3_^-^ channels also contribute to the influx of ionic compounds together with aquaporins for water influx to increase the turgor of the guard cell (Daszkowska-Golec and Szarejko, 2013). In a third scenario, the relative concentrations of the accumulation of organic acids such as malate and fumarate might regulate stomatal opening (Araújo et al., 2011a). Increased accumulation of malate (or fumarate) concentrations in the leaf mesophyll tissues, particularly in the apoplast, can promote stomatal closure by affecting adjacent guard cells (Araújo et al., 2011b). However, this scenario is unlikely as it is inconsistent with the current results from all the carboxylation module enzymes (and McPPCK), which showed a consistently strong correlation between elevated TA measurement and increased stomatal conductance. This scenario is also not supported for the NADP- and NADP-ME overexpression lines of the decarboxylation module, which showed a strong correlation between reduced TA measurements and lower rates of stomatal conductance.

Within the carboxylation module, the McBCA2-sGFP overexpression line, displayed significantly increased stomatal conductance, but no corresponding increase in TA (Figure 7). The increase in stomatal conductance resembled the small increases in stomatal conductance observed in transgenic tobacco plants expressing a cytosolic-localized version of BCA from tobacco (Majeau et al., 1994). In this instance, stomatal conductance is likely to be the result of lowering internal leaf CO_2_ concentrations through the formation of HCO_3_^-^ without any corresponding change in malate accumulation. The *35S::McPEPC1-sGFP*, *35S::McPPCK1-sGFP*, and the *35S::McNAD-MDH1-sGFP*, and *35S::McNAD-MDH2-sGFP* expressing lines all showed significantly increased TA that accompanied the increased stomatal conductance. These results suggest that each of these enzymes (or regulatory protein kinase), can promote malate formation in *A. thaliana*. However, the *35S::McNADP-MDH1-sGFP* overexpression line did not exhibit elevated TA, presumably because this enzyme, which is localized to the chloroplast, is less directly linked to the flow of carbon into C_4_ acids.

The observed increases in titratable acidity and likely malate accumulation in the McPEPC overexpression lines of *A. thaliana* is similar to the increased titratable acidity and malic acid accumulation observed in ZmPEPC overexpression lines of tobacco relative to wild-type plants (Hudspeth et al., 1992). Expression of the ZmPEPC in transgenic tobacco resulted in up to a 1.5-fold increase in malate accumulation (Kogami et al., 1994), but no growth stimulation was observed in these lines. No effect of ZmPEPC overexpression on CO_2_ assimilation rates, photosynthetic rates, or CO_2_ compensation points was observed. Similarly, overexpression of a maize C_4_-PEPC in transgenic rice plants had no discernable effect on stomatal opening and no positive effects on photosynthetic CO_2_ fixation (Fukayama et al., 2003). These results were also similar to transgenic potato expressing a bacterial form of PEPC isolated from *Corynebacterium glutamicum* compared with wild-type plants, which was thought to arise from the recapture of photorespiratory CO_2_ (Gehlen et al., 1996; Häusler et al., 1999; Häusler et al., 2002). Stomatal opening was accelerated in these plants compared with anti-sense lines with reduced PEPC expression (Gehlen et al., 1996). These results also resembled the increased malate accumulation reported for potato plants overexpressing a C_4_-photosynthesis-like PEPC from potato (*Solanum tuberosum*) (Rademacher et al., 2002). The expression of this modified form of PEPC under the control of a dark-inducible (DIN10) or a strong constitutive promoter (CaMV 35S) in *A. thaliana* resulted in a marked increase in stomatal conductance, transpiration, and dark respiration compared to wild-type plants (Kebeish et al., 2012). These modified PEPC overexpressing plants also exhibited incremental improvements in CO_2_ assimilation rates compared to wild-type controls.

In contrast to the effects observed for the carboxylation module enzymes, all four of the NAD-ME and NADP-ME overexpression lines of the decarboxylation module showed significant ∼1.3-fold decreases in stomatal conductance and TA accumulation, presumably because of malate decarboxylation, which is presumed to result in the direct release of CO_2_ increasing internal leaf CO_2_ concentrations within the leaf to drive stomatal closing, while decreasing malate accumulation (Figure 7). Previous examples of overexpression of NAD(P)-ME showed results consistent with those observed in the present study. For example, the overexpression of a chloroplast-targeted C_4_ NADP-ME from maize in tobacco resulted in reduced stomatal conductance and improved WUE compared with wild-type plants, but led to leaf necrosis in lines with high NADP-ME expression (Laporte et al., 2002). In another example, the overexpression of a chloroplast-localized C_4_ NADP-malic enzyme from maize in *A. thaliana* resulted in decreased malate and fumarate content (Fahnenstich et al., 2007). In this instance, the decreased organic acid (i.e., malate and fumarate) accumulation resulted in early senescence during growth under extended dark conditions (Fahnenstich et al., 2007). The underlying cause of the observed leaf necrosis or early leaf senescence was likely a result of the consumption of these metabolites in the dark, which was confirmed in a later study and highlights the important roles of malate and fumarate as essential carbon storage molecules in *A. thaliana* (Zell et al., 2010). In the current study, the rosette and leaf morphology of each of the C_4_-metabolism gene overexpression lines appeared normal except for apparent variations in size. No lines exhibited any notable leaf necrosis or early senescence under the growth conditions used.

The overexpression of McPPDK1 and McPPDK-RP in *Arabidopsis* failed to cause significant changes in stomatal conductance and TA, presumably because these enzymes are not involved directly in the release of CO_2_ within the leaf and in malate production. Interestingly, the overexpression of McPEPCK, which also involves the release of CO_2_ during the conversion of OAA to PEP, did not result in a significant change in stomatal conductance or TA (Figure 7A). This lack of phenotype probably occurred because there was no significant increase in OAA formation supplied by an accompanying NAD(P)-MDH activity. Such a lack of increased OAA flux likely prevented any significant increase in CO_2_ release by *McPEPCK*, which would explain the lack of stomatal closure in this line. However, additional daytime or continuous 24-h stomatal conductance measurements should be undertaken to confirm that overexpression of these C_4_-cycle components are not impacting stomatal behavior in some way. In *A. thaliana*, knockout mutants of AtPEPCK1, which is expressed specifically in guard cells and trichomes, displayed increased stomatal conductance and apertures and impaired stomatal closure in the dark presumably through alterations in malate metabolism (Penfield et al., 2012). Overall, the observed increases in increased stomatal conductance with accompanying increases in TA were consistent with either an increase in a reduction in intracellular CO_2_ and malate accumulation that promote stomatal opening in the case of the carboxylation module enzymes (except *McBCA2*) or an increase in intracellular CO_2_ and a reduction in malate formation that can drive stomatal closure in the case of the NAD- and NADP-ME of the decarboxylation module.

## Conclusion

Here we have laid a solid foundation for CAM Biodesign by characterizing the expression, subcellular localization, and functional phenotypes derived from the overexpression of 13 enzymes and regulatory proteins of the core C_4_-metabolism cycle of CAM in the C_3_-photosynthesis model species, *A. thaliana*. Although this report is an important first step, CAM plants are characterized by far more than the relatively simple biochemistry and regulatory steps present within the C_4_-cycle. Given the energetic cost of performing CAM (Shameer et al., 2018), the development of a water-deficit-inducible CAM expression system with optimal, mesophyll-specific and time-appropriate, diel or circadian regulatory elements would be preferred so that the water-saving effects of CAM are triggered only when demanded by proximate growing conditions (Borland et al., 2014). In addition, changes in carbohydrate transport, storage, and degradation are necessary to accommodate the diel provisioning of PEP for nocturnal CO_2_ uptake and organic acid production also occur in CAM species and these would have to be better understood and engineered to optimize CAM Biodesign efforts (Borland et al., 2016). In addition to inverse stomatal behavior that improves WUE, CAM species display a diverse suite of functional attributes, such as anatomical adaptations for succulence that allows for C_4_-acid and soluble sugar storage and the attenuation of drought, as well as tolerance to heat, high light intensities, UV-B irradiation, and photosynthetically active surfaces (Borland et al., 2009). Thus, continuing CAM Biodesign efforts might have to be combined with at least some consideration for increased cell size and vacuolar organic acid storage capacity (Lim et al., 2018) and optimized leaf anatomy (Zambrano et al., 2014). Nonetheless, the characterization of the key component of the core C_4_-metabolism CAM represents a critical first step in laying the foundation for ongoing CAM Biodesign efforts involving the construction of gene circuits consisting of discrete carboxylation and decarboxylation modules and various combinations of complete C_4_-cycle gene circuits.

## Data Availability Statement

The cDNA sequences generated for this study can be found in the ice plant (*Mesembryanthemum crystallinum*) Transcriptome Shotgun Assembly (TSA) project that has been deposited at DDBJ/EMBL/GenBank under the accession GBLK01000000 – GBLK01024204 (https://www.ncbi.nlm.nih.gov/nuccore/GBLK01000000).

## Author Contributions

JC conceived of the overall study. SDL, WY, and JC co-wrote the manuscript. WY curated and analyzed all transcript abundance data and assisted with gene selection. SDL prepared all protein fusion constructs and created all transgenic *A. thaliana* lines. SDL, SL, and W-GC collected and analyzed the phenotypic data from the transgenic *A. thaliana* lines. All authors read and approved the final manuscript.

## Conflict of Interest Statement

The authors declare that the research was conducted in the absence of any commercial or financial relationships that could be construed as a potential conflict of interest.

## Footnotes

1 http://arthropods.eugenes.org/EvidentialGene/

2 http://imagej.nih.gov/ij/

## Supplementary Material

Figure S1 Overexpression of individual carboxylation/decarboxylation module ice plant CAM genes alters plant biomass in *Arabidopsis*. T_3_ homozygous seeds of multiple lines of *35S::sGFP* Empty Vector (EV) control, *35S::McBCA2-sGFP*, *35S::McPEPC1-sGFP*, *35S::McPPCK1-sGFP*, *35S::McNAD-MDH1-sGFP*, *35S::McNAD-MDH2-sGFP*, *35S::McNADP-MDH1-sGFP*, *35S::McNAD-ME1-sGFP*, *35S::McNAD-ME2-sGFP*, *35S::McNADP-ME1-sGFP*, *35S::McNADP-ME2-sGFP*, *35S::McPPDK-sGFP*, *35S::McPPDK-RP-sGFP*, and *35S::McPEPCK-sGFP* were germinated and grown in soil mix under a 12-h photoperiod. Four-week-old plants were used to analyze overall plant biomass. **(A)** Quantification of rosette diameter (*n* = 10). **(B)** Quantification of leaf area (*n* = 20). **(C)** Quantification of leaf fresh weight (FW) (*n* = 20). Values represent means ± SD, ns, non-significant, ^∗^*p* < 0.05, ^∗∗^*p* < 0.01, and ^∗∗∗^*p* < 0.001, One-way ANOVA with Dunnett’s multiple comparison test.

Table S1 List of CAM gene-cloning primers used in this study.
