# Supplementary material for: Laying the Foundation for Crassulacean Acid Metabolism (CAM) Biodesign: Expression of the C4 Metabolism Cycle Genes of CAM in Arabidopsis
Source: Front Plant Sci. 2019 Feb 11;10:101. doi: 10.3389/fpls.2019.00101 (PMC6378705; doi:10.3389/fpls.2019.00101)
Supplement: Figure S1 — Overexpression of individual carboxylation/decarboxylation module ice plant CAM genes alters plant biomass in Arabidopsis. T3 homozygous seeds of multiple lines of 35S::sGFP Empty Vector (EV) control, 35S::McBCA2-sGFP, 35S::McPEPC1-sGFP, 35S::McPPCK1-sGFP, 35S::McNAD-MDH1-sGFP, 35S::McNAD-MDH2-sGFP, 35S::McNADP-MDH1-sGFP, 35S::McNAD-ME1-sGFP, 35S::McNAD-ME2-sGFP, 35S::McNADP-ME1-sGFP, 35S::McNADP-ME2-sGFP, 35S::McPPDK-sGFP, 35S::McPPDK-RP-sGFP, and 35S::McPEPCK-sGFP were germinated and grown in soil mix under a 12-h photoperiod. Four-week-old plants were used to analyze overall plant biomass. (A) Quantification of rosette diameter (n = 10). (B) Quantification of leaf area (n = 20). (C) Quantification of leaf fresh weight (FW) (n = 20). Values represent means ± SD, ns, non-significant, ∗p < 0.05, ∗∗p < 0.01, and ∗∗∗p < 0.001, One-way ANOVA with Dunnett’s multiple comparison test. [file Data_Sheet_1.docx]

Supplementary Material

**Laying the foundation for crassulacean acid metabolism (CAM) Biodesign: Expression of the C_4_ metabolism cycle genes of CAM in *Arabidopsis*.**

Sung Don Lim^1^, Sojeong Lee^1^, Won-Gyu Choi^1^, Won Cheol Yim^1^, and John C. Cushman^1,*^

^1^Department of Biochemistry and Molecular Biology, University of Nevada, Reno, Reno, NV, USA.

***Correspondence:**

Professor John C. Cushman

[jcushman@unr.edu](mailto:jcushman@unr.edu)
