# Supplementary material for: Laying the Foundation for Crassulacean Acid Metabolism (CAM) Biodesign: Expression of the C4 Metabolism Cycle Genes of CAM in Arabidopsis
Source: Front Plant Sci. 2019 Feb 11;10:101. doi: 10.3389/fpls.2019.00101 (PMC6378705; doi:10.3389/fpls.2019.00101)
Supplement: Table S1 — List of CAM gene-cloning primers used in this study. [file Table_1.docx]

Supplementary Material

**Laying the foundation for crassulacean acid metabolism (CAM) Biodesign: Expression of the C_4_ metabolism cycle genes of CAM in *Arabidopsis*.**

Sung Don Lim^1^, Sojeong Lee^1^, Won-Gyu Choi^1^, Won Cheol Yim^1^, and John C. Cushman^1,*^

^1^Department of Biochemistry and Molecular Biology, University of Nevada, Reno, Reno, NV, USA.

***Correspondence:**

Professor John C. Cushman

[jcushman@unr.edu](mailto:jcushman@unr.edu)

**Supplementary Table 1.** List of CAM gene-cloning primers used in this study.

| **Name** | **Forward primer (5' to 3')** | **Reverse primer (5' to 3')** |
| --- | --- | --- |
| *McBCA2* | CACCATGACAGGAGGCTTTAGGA | TACTGCGGTAGAAGGTGAGAG |
| *McPEPC1* | CACCATGTCGACTGTGAAGCTAGA | ACCAGTGTTCTGCAGACCA |
| *McPPCK1* | CACCATGTGTGAGAGCTTCAAGAG | CATGTTGGCCAATCCTC |
| *McNAD-MDH1* | CACCATGGCCGTTGAACCTCT | AGTCAGGCATGAGTAGGCC |
| *McNAD-MDH2* | CACCATGAGGTCCCAGTTGCTGAG | ATTCTTGTTGGCAAAGTCGATT |
| *McNADP-MDH1* | CACCATGGCAGTGGCAGAGCTTTC | CATTTCTCCTGGAAGCATTGT |
| *McNAD-ME1* | CACCATGTCGATGATGATCCATCG | GAGGTTGATGTGACGAAGCTTATA |
| *McNAD-ME2* | CACCATGTGGAGGCTTACGCGATC | TTTCTCATGAACTAGCGGGC |
| *McNADP-ME1* | CACCATGGGTGGTAGCAATGCAT | ACGGAAACTCCGGTAAG |
| *McNADP-ME2* | CACCATGATCTCCTTACACAGAGCCAA | CCGGTAGGTTCTGTAGGCAG |
| *McPPDK* | CACCATGGCGTCAGCTTTCAAGG | AACGACTTGAGCTGCTG |
| *McPPDK-RP* | CACCATGTTAGCTTGTGCTAGC | ATAGCGCTTAGATATTCTGGGC |
| *McPEPCK* | CACCATGGCGGAAAACAACGGG | GAAATTAGGACCGGCTGCA |
